# Small Cell Carcinoma of the Uterine Cervix in a Pregnant Patient Diagnosed with Liquid Based Cytology and Cell Block Immunocytochemistry

**DOI:** 10.1155/2014/971464

**Published:** 2014-08-28

**Authors:** Mawuli F. Attipoe, Charles D. Sturgis

**Affiliations:** ^1^Duke University, Durham, NC 27708, USA; ^2^CellNetix Pathology and Laboratories, Everett, WA 98201, USA; ^3^Providence Regional Medical Center Everett, Everett, WA 98201, USA; ^4^The Everett Clinic, Everett, WA 98201, USA; ^5^Cleveland Clinic, Robert J. Tomsich Pathology Institute, 9500 Euclid Avenue L25, Cleveland, OH 44195, USA

## Abstract

Definitive cytomorphologic diagnosis of small cell carcinoma of the uterine cervix is possible but can be challenging in routine cervicovaginal cancer screening specimens. Several small series of reported cases of cervical small cell carcinoma have shown this uncommon malignancy to represent fewer than 2% of all invasive cervical cancers. This tumor type is associated with poor prognosis and rapid disease progression and can develop to an advanced stage in the interval between screening visits. Only rare case reports of small cell carcinoma arising in gravid cervices are known. In the current case a 29-year-old, gravida 6, para 2, pregnant (10-week gestation) female presented with postcoital bleeding. A definitive diagnosis of small cell carcinoma of the cervix was made possible by liquid based Pap testing with ancillary cell block preparation allowing for immunocytochemical characterization of the lesional cell population.

## 1. Introduction

The current World Health Organization (WHO) classification of tumors of the female genital organs describes four uncommon types of primary cervical neuroendocrine neoplasms. These include carcinoid tumor, atypical carcinoid tumor, large cell neuroendocrine carcinoma, and small cell carcinoma [1]. Perhaps the most clinically aggressive of these tumor types is small cell carcinoma with afflicted patients often showing rapid disease progression and poor outcomes [2–7]. Several studies have shown small cell carcinoma of the uterine cervix to represent fewer than 2% of all invasive cervical cancers [8, 9]. Some small series and individual case reports of patients with cervical small cell carcinoma are known in the literature; however, only very rare reports of this disease being initially diagnosed in gravid cervices are known [10–13]. Establishing a definitive diagnosis of this aggressive malignancy by cervicovaginal cytology testing alone can be especially challenging, as ancillary studies (such as immunocytochemistry) are commonly employed in confirming a specific diagnosis [14–18]. Herein we report a case of uterine cervical small cell carcinoma diagnosed in a 29-year-old, pregnant female who presented with postcoital bleeding. As part of her evaluation, she underwent liquid based Pap testing with the additional step of cell block preparation allowing for immunocytochemical characterization of the lesional cell population and definitive diagnosis.

## 2. Case Presentation

A 29-year-old, gravida 6, para 2, adult female with a past medical history of scoliosis presented to a local walk-in clinic with a complaint of vaginal bleeding after intercourse the night before. An office lab test confirmed that the patient was pregnant. An examination performed by the walk-in clinic primary care doctor revealed an erythematous, swollen cervix with a small polypoid mass lesion. An ultrasound was performed and revealed an estimated fetal gestational age of 10 weeks 2 days. The patient was advised to abstain from intercourse and to seek obstetric consultation.

One month later, at her first true prenatal clinical exam, the patient indicated ongoing vaginal bleeding. She also commented on interval treatment with oral antibiotics for presumed bacterial vaginitis. Her cervicovaginal cytology history included a patient reported result of an “abnormal Pap” from greater than 10 years before. In addition, the electronic medical record at the institution in which she was receiving her obstetric care showed a Pap result of negative for intraepithelial lesion (NILM) with a positive Hybrid Capture II High-Risk HPV DNA test result from 17 months before. The polypoid lesion extending from the cervical os was again noted. A liquid based (ThinPrep) cervicovaginal cytology sample was obtained. The patient was again advised to abstain from intercourse.

Pap results confirmed a malignant neoplasm consistent with small cell carcinoma. The Pap interpretation was based upon the combination of cytomorphology, cell block cytomorphology, and ancillary immunocytochemical studies. A colposcopy was performed. In addition to a small exophytic mass, the colposcopy of the gravid cervix also revealed areas of acetowhite epithelium, areas of apparent necrosis, and atypical vasculature. The grossly evident lesion was biopsied. Histopathologic studies with additional immunocytochemical characterization confirmed the cytologic impression of small cell carcinoma. The patient was informed of the diagnosis and was referred to a regional gynecological oncology center. Within two weeks she presented to the local emergency department with complaints of abdominal pain and vaginal bleeding. An ultrasound revealed severe oligohydramnios, and the patient subsequently spontaneously miscarried. Following this, she underwent total abdominal hysterectomy and bilateral salpingo-oophorectomy at the regional center. Her disease process was again histologically confirmed as small cell carcinoma of the uterine cervix and was clinically and pathologically staged as FIGO IB2.

## 3. Discussion

Small cell carcinoma of the uterine cervix is rare and accounts for approximately 2% of all invasive cervical malignancies [8, 9]. Women with this disease process have been reported to range in age from 25 to 87 years with a median age of roughly 42 years [2, 4, 7, 8]. Because small cell carcinomas are biologically aggressive and rapidly growing, patients commonly present with symptoms rather than when asymptomatic during routine cervicovaginal screenings. In the uterine cervix, this uncommon disease process is known to be associated with concomitant human papillomavirus (HPV) infection. Scholarly articles on the topic of HPV association with cervical small cell carcinogenesis have shown variable percentages of cases to be HPV related [19–21]. Reported HPV incidence rates in such patients have ranged from 68% to 80% and higher. Most studies implicate HPV viral types 16 and 18 with HPV 18 appearing to be the most commonly associated viral type in cervical small cell carcinoma patients [19–21].

**Figure 1:**
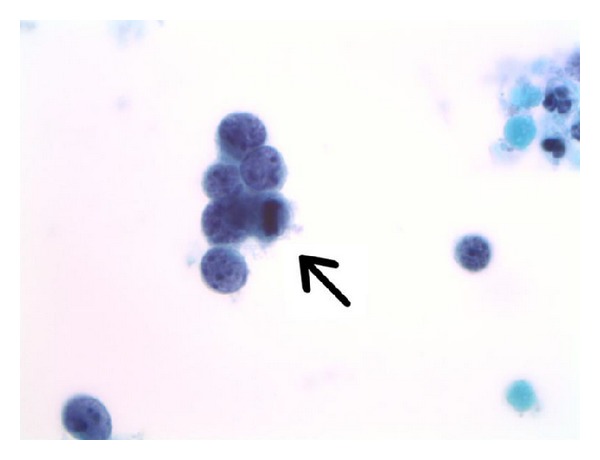
Lesional cells are present in cohesive aggregates as well as in singly dispersed fashions. These cells have scant, delicate cytoplasm and high nuclear/cytoplasmic ratios. The chromatin is finely granular without macronucleoli. A metaphase mitosis is noted (arrow) (Papanicolaou stain, ThinPrep, 1000x).

**Figure 2:**
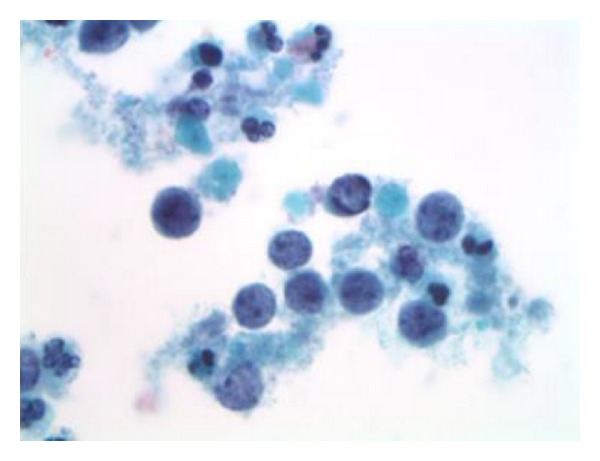
The individual malignant cells are small in size (not larger than twice the size of internal control segmented neutrophils). Areas of granular proteinaceous diathesis material are evident clinging to the lesional cells. Single necrotic cells (apoptotic bodies) are also observed (Papanicolaou stain, ThinPrep, 1000x).

**Figure 3:**
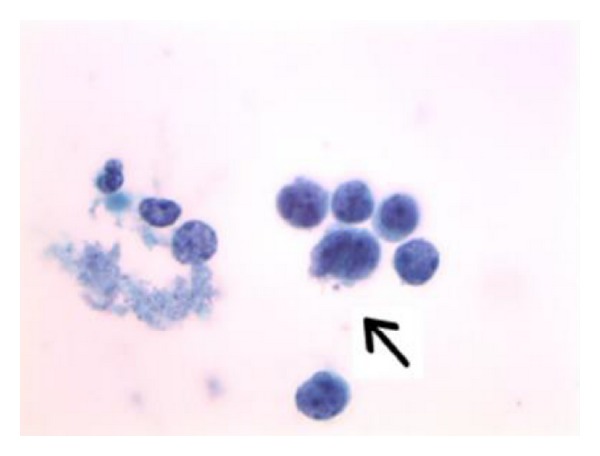
Intact tumor cells show delicate rims of amphophilic cytoplasm. Focal nuclear molding is seen (arrow) (Papanicolaou stain, ThinPrep, 1000x).

It is not uncommon for women with small cell carcinoma of the uterine cervix to have a prior Pap test history of negative for intraepithelial lesion or malignancy (NILM), as the disease process is classically rapidly progressive and can develop to an advanced state in the interval between cervicovaginal cytology screenings. Some patients with invasive small cell carcinoma can have a prior history of and/or concomitant findings of squamous intraepithelial lesions (SIL). As was the case for this patient, Pap test slides from small cell carcinoma patients are most often moderately to highly cellular [22]. The ThinPrep slide in this case showed as many or more lesional cells than normal squamous epithelial cells. The lesional cells were seen in loosely cohesive multidimensional aggregates and sheets as well as in singly dispersed fashions. The cells were monotonous in size, being approximately two times the size of internal control intermediate squamous cell nuclei. The lesional cells showed very high nuclear/cytoplasmic ratios with delicate rims of amphophilic cytoplasm, and mitoses were evident (Figure 1). The slide background was clear between cell groups, but in some areas granular proteinaceous diathesis material (clinging diathesis) was seen, and apoptotic degenerated single tumor cells were noted (Figure 2). The cells showed finely granular chromatin, and in focal areas nuclear molding was identified (Figure 3).

**Figure 4:**
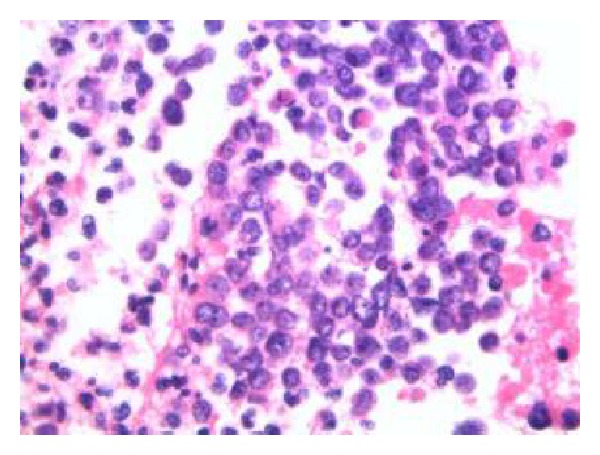
Microscopic image of a highly cellular cell block showing large aggregates of malignant cells. The cells are morphologically similar to those seen in the liquid based cytology with high nuclear/cytoplasmic ratios and finely granular chromatin. No cytoplasmic orangeophilia or evidence of keratinization is discerned. Admixed apoptotic bodies, necrotic granular debris, and inflammatory cells are present (hematoxylin and eosin stain, Cell Block, 630x).

Small cell carcinomas are cytologically similar at various body sites and are most commonly encountered in the lungs. Prior detailed regression analyses of thoracic small cell carcinomas have proven three specific cytomorphologic criteria (nuclear molding, finely granular/“salt and pepper” chromatin, and scant delicate cytoplasm) to be the most reliable features for separating small cell carcinomas from non-small-cell carcinomas [18]. In this case the cytomorphologic features were consistent with small cell carcinoma; however, other differential diagnostic considerations included lymphoma, small blue cell sarcomas, melanoma, and possibly other entities. Because of the need for additional testing to better characterize the cell type, a cell block was prepared from the residua of the liquid based Pap test sample (Figure 4).

Ancillary immunocytochemical studies were performed on the cell block. These tests included CD45, CD20, CD3, S-100, HMB45, pancytokeratin, CAM5.2, EMA, CK7, CK20, synaptophysin, chromogranin, p16, desmin, and CD99. Of these, p16, chromogranin, synaptophysin, and pancytokeratin showed positive immunoreactivity.

**Figure 5:**
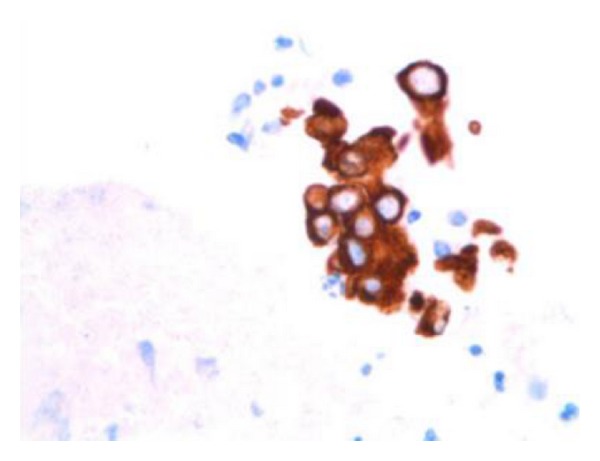
Intense strong cytoplasmic immunoreactivity is present with pancytokeratin (PanCK immunocytochemistry, Cell Block, 630x).

**Figure 6:**
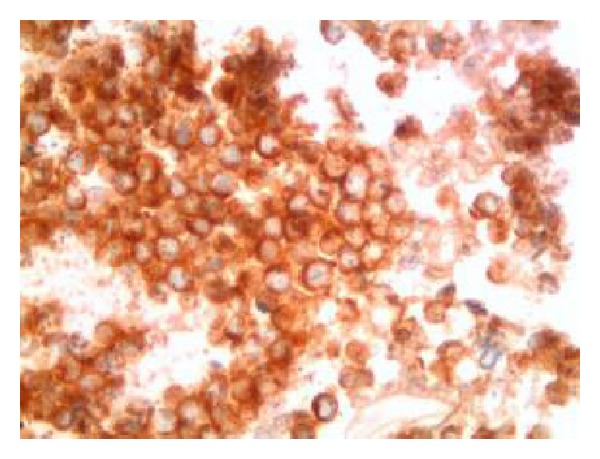
Malignant cells display uniform and diffuse cytoplasmic immunoreactivity with synaptophysin, supporting a neuroendocrine phenotype (synaptophysin immunocytochemistry, Cell Block, 630x).

**Figure 7:**
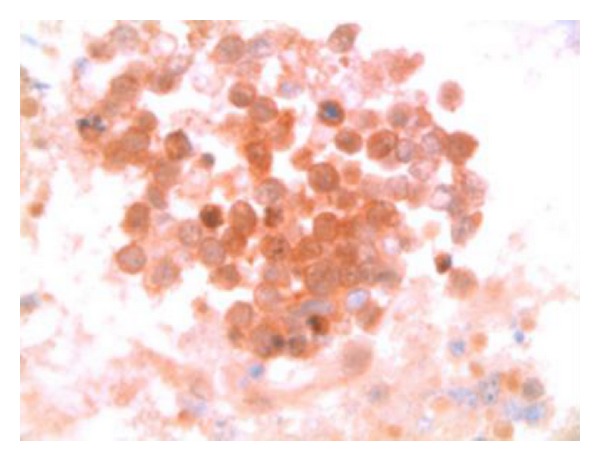
Microscopic image of lesional cells showing both cytoplasmic and nuclear reactivity with p16, suggesting an HPV mediated disease process (p16 immunocytochemistry, Cell Block, 630x).

The pancytokeratin study demonstrated strong cytoplasmic immunoreactivity, helping to confirm an epithelial origin (Figure 5). Synaptophysin reactivity was seen in the majority of intact cells with strong cytoplasmic positivity noted, suggesting a neuroendocrine phenotype (Figure 6). The p16 study showed both cytoplasmic and nuclear reactivity, possibly implicating an HPV mediated disease process (Figure 7). Of note a prior Pap test from this patient (performed 17 months before) had been interpreted as NILM but had shown a positive high risk HPV DNA result (QIAGEN, Hybrid Capture II). Negative CD45, CD20, and CD3 results assisted in excluding lymphoma as a possible diagnosis. S-100 and HMB45 were nonreactive, excluding melanoma. Desmin was similarly negative, helping to exclude the differential diagnosis of rhabdomyosarcoma. The negative CD99 study made a primitive neuroectodermal tumor (PNET) unlikely.

Subsequently, the patient underwent colposcopic biopsy with histologic confirmation of the diagnosis of small cell carcinoma of the uterine cervix. She later spontaneously miscarried and underwent definitive surgical management confirming a FIGO IB2 stage disease process. A follow-up ThinPrep Pap test conducted 5 months after the original diagnostic cytology study was NILM with reactive changes associated with inflammation.

Small cell carcinoma of the cervix is a potentially deadly disease. Prognosis is tied to stage at presentation as well as to the patient's baseline state of health. Definitive diagnosis in cervicovaginal cytology can be challenging. Cell block preparation (when feasible) may allow for ancillary testing and histomorphologic analyses. Definitive diagnosis is possible. Consideration for such ancillary cell block testing may be especially warranted in women who are not surgical candidates, especially in those who are pregnant.
